# Genetic and environmental influences in autism: guiding the future of tailored early detection and intervention

**DOI:** 10.1172/JCI201157

**Published:** 2025-11-17

**Authors:** Alexandra L. Bey, Scott Soderling, Geraldine Dawson

**Affiliations:** 1 Department of Psychiatry and Behavioral Sciences,; 2 Department of Cell Biology, and; 3 Department of Psychiatry and Behavioral Sciences, Duke University School of Medicine, Durham, North Carolina, USA.

## What causes autism?

Autistic individuals, families, clinicians, government officials, and policymakers have a great interest in understanding the causes of autism. This Viewpoint summarizes what is currently known about the etiological factors associated with autism and how this information is transforming early detection and intervention. Autism is now understood not as a single condition, but as a group of neurodevelopmental conditions that affect social interaction and communication and are characterized by restricted and repetitive behaviors. Autism varies widely, often accompanied by medical, developmental, and psychiatric co-occurring conditions.

**Figure 1:**
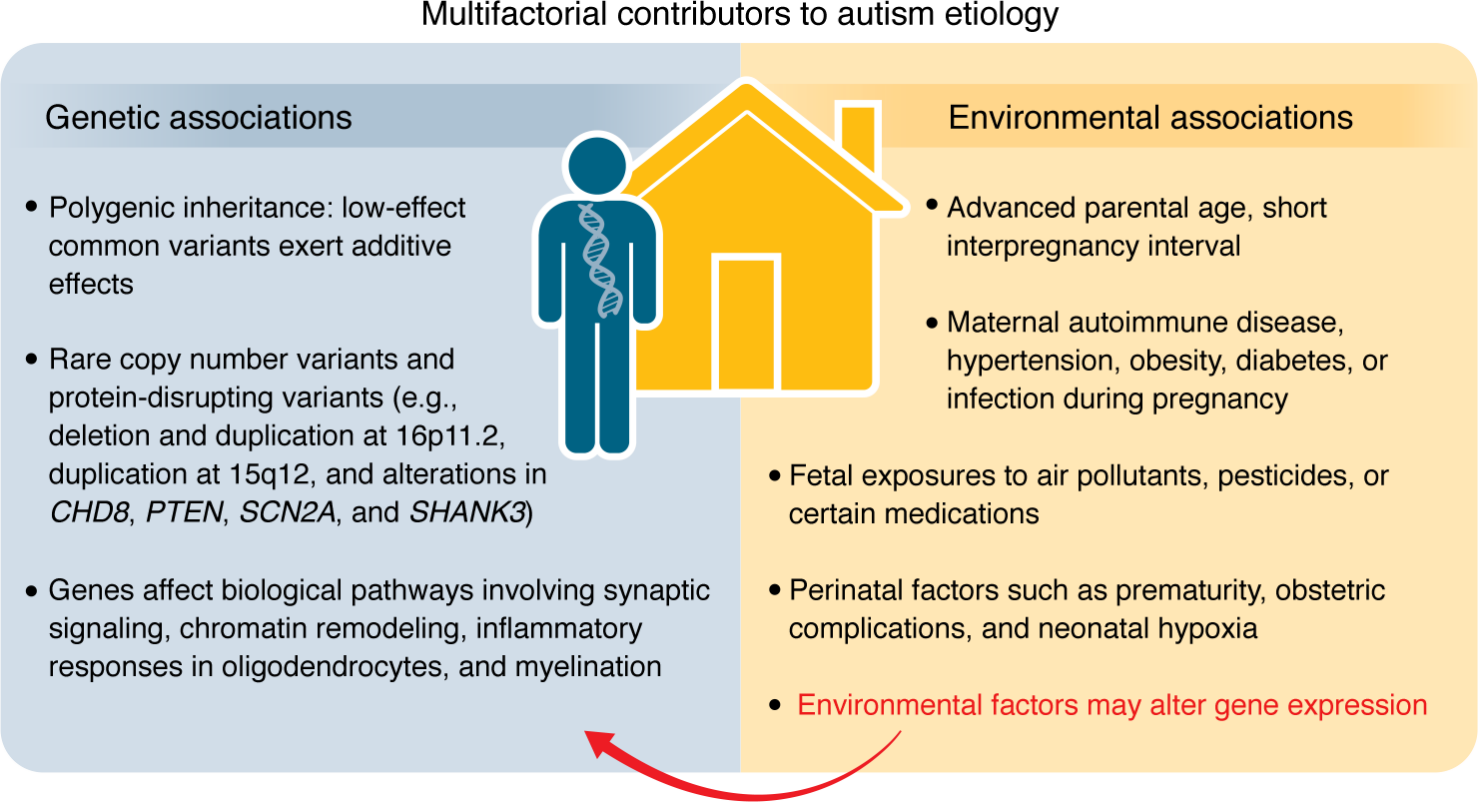
Summary of genetic and environmental factors associated with autism etiology.

Autism’s etiology is multifactorial, involving both genetic and environmental influences (Figure 1) (1). Hundreds of genes increase the likelihood of autism, with heritability estimates of approximately 80% based on family studies. Genes associated with autism are highly expressed during fetal brain development and converge on biological pathways involving synaptic signaling, chromatin remodeling, inflammatory responses in oligodendrocytes, and myelination (1).

The combined effects of rare genetic variants and polygenic liability contribute to autism (2). Rare inherited and spontaneous genetic mutations are identified in a subgroup of autistic individuals. These include copy number variants and protein-disrupting variants (e.g., deletion and duplication at 16p11.2, duplication at 15q12, and alterations in *CHD8*, *PTEN*, *SCN2A*, and *SHANK3*). These mutations are not exclusive to autism and are also associated with intellectual disability, epilepsy, and motor dysfunction. Most autism cases are related to common inherited genes of low individual effect that exert additive effects in a polygenic manner. These common polygenetic influences are thought to be more specific to autism’s core features.

Environmental factors also increase autism likelihood, accounting for approximately 40% of variance in twin studies (3, 4). Familial factors include advanced parental age; short interpregnancy interval; and maternal autoimmune disease, hypertension, obesity, diabetes, or infection during pregnancy (3). Fetal exposures to air pollutants, pesticides, or medications such as valproate or selective serotonin reuptake inhibitors have also been associated with autism, although no association is found for the latter when maternal psychiatric conditions are accounted for (3). Some studies have linked autism to prenatal exposure to acetaminophen; however, a recent study of over 2 million children found no association when familial factors were controlled (5). Prenatal folic acid supplementation is associated with decreased autism likelihood and may ameliorate the impacts of neurotoxicants (3). Finally, perinatal factors such as prematurity, obstetric complications, and neonatal hypoxia are associated with autism and may mediate maternal factors (6).

Thus, autism arises from a wide range of influences on prenatal brain development. A threshold susceptibility model proposes that rare and common genetic variants, combined with environmental factors, contribute to autism. Understanding these diverse influences is essential for advancing autism subtyping and personalized approaches to diagnosis and intervention.

## Autism stratification approaches and diagnostic biomarker discovery

### Advances in genetic profiling, stratification, and diagnostic biomarkers.

Research on etiological factors associated with autism is shaping clinical care. A key goal is to provide behavioral and medical intervention as early as possible to reduce the disabilities often associated with autism. Genetic testing is considered standard of care for individuals with autism, although in practice, rates of clinical genetic testing are low (1, 7). A genetic diagnosis can lead to targeted treatments (e.g., dietary modification for inborn errors of metabolism), syndrome-specific recommendations (e.g., practice parameters for Rett Syndrome or Phelan-McDermid Syndrome), and clinical trial opportunities. The diagnosis helps families plan for future support and medical care, since conditions such as intellectual disability, heart conditions, gastrointestinal problems, and seizures differ by genetic etiology.

For autistic individuals without an identifiable genetic etiology, a major challenge is to identify biological subgroups to provide personalized intervention approaches. Generative mixture modeling has been used to link phenotypic subgroups of autism and clinical outcomes with genetic and molecular pathways tied to common de novo and inherited variation (8). Distinct genetic sets and pathways aligned with subgroups differing in autistic traits, cognitive impairment, and psychiatric conditions. Phenotypic subgroup–specific differences in the developmental timing of affected genes corresponded with clinical outcomes, suggesting the potential for more precise diagnosis and early clinical guidance. Another study identified two subgroups among autistic individuals based on polygenic factors: the first was characterized by earlier diagnosis and lower social and communication abilities, whereas the second group was characterized by later autism diagnosis and socioemotional and behavioral difficulties in adolescence (9).

Researchers are also working to identify infants with a higher autism likelihood before a behavioral diagnosis is possible at 18–24 months. Longitudinal studies of infant siblings — who have increased genetic susceptibility for autism — identified prediagnostic biomarkers using MRI, electrophysiology, and eye-tracking metrics (10). MRI studies show altered connectivity in the cerebellum, splenium, corpus callosum, auditory and somatosensory regions, amygdala, anterior temporal cortex, and ventral-medial prefrontal cortex. Event-related potentials (ERPs) to faces, eye gaze, phonemes, and sensory stimuli are also altered in infants who are later diagnosed with autism (10). Prediction of autism diagnosis based on infant ERPs to faces was improved when combined with polygenetic scores (11). Machine learning applied to electronic health records has shown promise for predicting autism during infancy, enabling prediagnostic screening, earlier diagnostic referrals, and timely access to interventions (12). Caregivers can be taught strategies to promote language and social development in infants with early signs of autism (13).

### Environmental stratification and biomarker development.

Environmental factors linked to autism are hypothesized to converge on elevated inflammation, oxidative stress, and altered hormone regulation during critical neurodevelopmental periods (3, 4). Biomarkers from blood, saliva, cerebrospinal fluid, or hair have shown weak or nonsignificant associations with autism. These include neurotransmitters, neurotrophic growth factors, inflammatory markers, hormones, and proxies for environmental exposures, including vitamins, heavy metals, or air pollutants. Studies are constrained by small sample sizes, limited sampling during key neurodevelopmental windows, high heterogeneity, and a lack of consideration of genetic factors.

Preliminary studies have identified environmental exposures influencing the likelihood of genetic mutations and variable susceptibility to environmental exposures based on genotype (3). Furthermore, environmental exposures may influence the expression of neurodevelopmental genes. Epigenetic biomarkers from parental, placental, or neonatal samples may enhance stratification for autism screening, diagnostics, and intervention.

## Progress in therapeutics discovery

### Harnessing genetic insights to guide novel autism therapies.

Emerging therapeutics include restoring gene function in cases of haploinsufficiency, reactivating silenced genes, and delivering small molecules acting on pathways downstream of affected genes (14). Following promising preclinical studies demonstrating that viral-mediated gene replacement in postnatal animals improves brain and behavioral phenotypes (15, 16), clinical trials are underway for the replacement of the methyl-CpG binding protein 2 (*MECP2*) gene in Rett syndrome and the SH3 and multiple ankyrin repeat domains 3 (*SHANK3*) gene in Phelan-McDermid syndrome. Regulation of gene dosage and correct spatiotemporal delivery are notable challenges in these approaches, as neurodevelopmental conditions can arise when genes are deleted *or* duplicated, as is the case for the *MECP2*, *SHANK3*, and ubiquitin-protein ligase E3A (*UBE3A*) genes (14). Approaches incorporating gene regulatory elements in viral constructs or targeting CRISPR/Cas9-mediated repair to the endogenous mutated allele are being explored in preclinical models (15).

Preclinical studies have demonstrated that reactivating a silenced allele improves neuronal functioning and behavior. In neurons, the maternal allele of *UBE3A* is expressed, whereas a noncoding RNA antisense transcript binds to the paternal allele and silences it. Using antisense oligonucleotides or CRISPR/Cas9 to disrupt the antisense transcript leads to unsilencing of the paternal allele of *UBE3A* and improvement of neuronal and behavioral phenotypes of Angelman syndrome model mice (17). Fragile X syndrome is caused by increased CGG repeats in the fragile X messenger ribonucleoprotein (*FMR1*) gene promoter, which leads to its silencing. Various approaches to alter FMR1 expression, whether through CRISPR/Cas9-mediated excision or recruiting endogenous DNA repair mechanisms, have demonstrated in vitro or in vivo preclinical efficacy (18). There are challenges related to the timing of delivery, given the likelihood that delivery needs to occur early in brain development.

Convergent molecular differences in genetic and idiopathic forms of autism are inspiring additional therapeutic approaches. Lower levels of the neurotrophic factor IGF-1 have been observed in some autistic individuals, and preclinical studies link environmental exposures such as maternal immune activation or perinatal hypoxia to reduced IGF-1 in mouse pups (19). Increasing IGF-1 leads to increased synaptic development and reduced neuroinflammation, two potential therapeutic targets in autism. In 2023, trofinetide, a synthetic analog of the IGF-1 N-terminus, was approved to treat Rett syndrome (20), with other trials underway for this or related compounds in Fragile X, Phelan-McDermid, Angelman, and Prader-Willi syndromes.

Researchers have also applied proteomics technologies to understand biological pathways in which autism genes participate and identify convergent molecular differences that may be leveraged for therapeutics (21–23). Together, three studies analyzed the proteins encoded by 60 different autism-related genes. Among the findings common to these studies was the association of autism-related genes with synaptic transmission pathways, as well as differential expression of genes in excitatory neurons of cortical layers 2/3 that represent a component of an autism-associated proteome. Each study identified smaller gene clusters within autism-associated pathways, suggesting convergence into subsets of functional pathways. Moreover, high-confidence autism gene products often associate with lower-confidence ones, supporting the need to prioritize lower-confidence genes in future studies. These findings support the polygenic etiology of autism, i.e., combinatorial interactions among genes of individually lower effect. Finally, each study demonstrated that specific mutations associated with autism alter the protein networks formed. These initial studies suggest that expanded analysis at the proteome level will uncover functional interactions among autism genes, pathways of biological convergence, and how missense mutations, exon skipping, and truncating variants alter these networks. The hope is that understanding mechanistically how autism genetic mutations affect these pathways may reveal new therapeutic targets, as supported by these studies (23).

### From environmental exposures to targets for intervention.

Growing knowledge of environmental factors associated with autism is driving new mechanistic studies and therapeutic approaches. Tools are being developed to screen chemicals for their impact on key genes and processes involved in neurodevelopment. These approaches integrate genetic and environmental data to better understand interactive effects. Approaches include screening for neurodevelopmental impacts of chemical combinations or assessing specific compounds in cellular or mouse models with autism-associated mutations (24). By studying the interactions between the maternal immune system and fetal neurodevelopment, new insights into how maternal infections during pregnancy increase autism likelihood may lead to more individualized screening and novel therapeutics targeting inflammatory pathways, such as blocking select cytokines (25).

## Challenges and future directions

New technologies and computational approaches offer promise for personalized therapies for autistic individuals with identifiable genetic variants. While no autism-related gene therapies are approved, progress is underway for several autism-related syndromes, with the goal of reducing disability associated with these syndromes. Preclinical in vitro and in vivo studies are shedding light on mechanisms underlying monogenic and syndromic autism. Looking to the future, modifying biological pathways impacted by autism-related variants to improve the quality of life for autistic individuals will be challenging. However, emerging deep-learning/artificial intelligence advances in protein engineering hold great promise for developing the tools that will be needed (26). Additionally, researchers are taking advantage of a variety of model systems (mouse and human neurons; in vitro and in vivo) to gain insights into mechanisms that account for the disabilities and medical conditions (e.g., seizures) associated with autism. Advances in organoids and assembloids that model human cell types during development provide additional approaches for discovering genetic and environmental triggers and platforms for testing strategies to modify these triggers or the response to them (27).

For polygenic forms of autism, a major challenge is the multifactorial nature of autism’s etiology and complex causal pathways. Stratifying autism into subgroups based on mechanism and phenotype – using multimodal methods that integrate genetic, environmental, imaging, and behavioral data — could help reclassify types of autism and predict clinical course and response to treatments. Clinical trials will benefit from stratification biomarkers to reduce heterogeneity. Initiatives such as the NIH Autism Biomarkers Consortium for Clinical Trials have identified EEG and eye-tracking biomarkers (28). However, autism’s phenotypic variability across individuals and domains of functioning presents challenges in identifying objective, quantitative screening tools and outcome measures for clinical trials that are sensitive to change. Digital phenotyping and wearable devices offer promising solutions (29). Choosing the appropriate outcome measure is an important consideration, as there are various domains of disability associated with autism, including cognitive and language impairment and medical conditions.

Autism is a spectrum: some people need constant, intensive care, while others live independently, work, and thrive with support. Early detection of autism through biomarkers could allow early intervention before a formal diagnosis can be made. Such advances in early autism detection and new therapies raise ethical questions about the rights and priorities of autistic individuals and their families. Safety, informed consent, cost, and availability are among the many ethical issues that need attention. Many autistic individuals see autism as a positive part of their identity and advocate for access to support and research that aligns with their priorities. Our efforts to develop better screening and diagnostic tools and therapies must recognize and address this diversity. Involving autistic individuals, caregivers, and providers in designing studies and developing screening tools and outcome measures will help ensure that new approaches to early detection and intervention translate into meaningful, long-term improvements in quality of life for autistic individuals and their families.

## Funding support

This work is the result of NIH funding, in whole or in part, and is subject to the NIH Public Access Policy. Through acceptance of this federal funding, the NIH has been given a right to make the work publicly available in PubMed Central.

NICHD, 2P50HD093074 (to GD).
NIMH, K23MH135224 (to ALB).
NIH, R01MH111684 (to SS).

## Version 1 11/17/2025

Electronic publication
